# Carcinogenic polycyclic aromatic hydrocarbons induce CYP1A1 in human cells via a p53-dependent mechanism

**DOI:** 10.1007/s00204-014-1409-1

**Published:** 2014-11-15

**Authors:** Laura E. Wohak, Annette M. Krais, Jill E. Kucab, Julia Stertmann, Steinar Øvrebø, Albrecht Seidel, David H. Phillips, Volker M. Arlt

**Affiliations:** 1 Analytical and Environmental Sciences Division, MRC-PHE Centre for Environment and Health, King’s College London, Franklin-Wilkins Building, 150 Stamford Street, London, SE1 9NH UK; 2 Section of Molecular Carcinogenesis, Institute of Cancer Research, Sutton, Surrey, UK; 3 Department of Biological and Chemical Working Environment, National Institute of Occupational Health, Oslo, Norway; 4 Biochemical Institute for Environmental Carcinogens, Prof. Dr. Gernot Grimmer-Foundation, Grosshansdorf, Germany

**Keywords:** Benzo[*a*]pyrene, Tumour suppressor p53, Cytochrome P450, Carcinogen metabolism, DNA adducts

## Abstract

The tumour suppressor gene *TP53* is mutated in more than 50 % of human tumours, making it one of the most important cancer genes. We have investigated the role of *TP53* in cytochrome P450 (CYP)-mediated metabolic activation of three polycyclic aromatic hydrocarbons (PAHs) in a panel of isogenic colorectal HCT116 cells with differing *TP53* status. Cells that were *TP53(+/+)*, *TP53(+*/−*)*, *TP53(−/−)*, *TP53(R248W/+)* or *TP53(R248W/−)* were treated with benzo[*a*]pyrene (BaP), dibenz[*a,h*]anthracene and dibenzo[*a,l*]pyrene, and the formation of DNA adducts was measured by ^32^P-postlabelling analysis. Each PAH formed significantly higher DNA adduct levels in *TP53(+/+)* cells than in the other cell lines. There were also significantly lower levels of PAH metabolites in the culture media of these other cell lines. Bypass of the need for metabolic activation by treating cells with the corresponding reactive PAH-diol-epoxide metabolites resulted in similar adduct levels in all cell lines, which confirms that the influence of p53 is on the metabolism of the parent PAHs. Western blotting showed that CYP1A1 protein expression was induced to much greater extent in *TP53(+/+)* cells than in the other cell lines. CYP1A1 is inducible via the aryl hydrocarbon receptor (AHR), but we did not find that expression of AHR was dependent on p53; rather, we found that BaP-induced CYP1A1 expression was regulated through p53 binding to a p53 response element in the *CYP1A1* promoter region, thereby enhancing its transcription. This study demonstrates a new pathway for *CYP1A1* induction by environmental PAHs and reveals an emerging role for p53 in xenobiotic metabolism.

**Electronic supplementary material:**

The online version of this article (doi:10.1007/s00204-014-1409-1) contains supplementary material, which is available to authorized users.

## Introduction

The *TP53* tumour suppressor gene, which encodes the protein p53, is mutated in over 50 % of human tumours and is one of the most important cancer genes (Olivier et al. 2010). p53, often described as the guardian of the genome, is involved in multiple cellular functions; amongst these is the response to cellular stress induced by various types of DNA damage, thereby delaying DNA synthesis or cell division to allow DNA repair or inducing apoptosis. In normal, unstressed cells, p53 protein expression is kept low via ubiquitin-mediated proteolysis that is regulated by the E3 ubiquitin ligase MDM2. Disruption of the normal p53 response by *TP53* mutation leads to the development of tumours. Besides acquired somatic mutations in the *TP53* gene being a common feature of the cancer genotype, germline mutations can cause predisposition to a wide spectrum of early onset cancers associated with the Li–Fraumeni and Li–Fraumeni-like syndromes (Olivier et al. 2010). Furthermore, some *TP53* polymorphisms in coding and noncoding regions have been shown to increase cancer susceptibility and to modify cancer phenotypes in *TP53* mutation carriers (Whibley et al. 2009). In addition to its role in the DNA damage response, p53 has also been found to regulate metabolic pathways such as glycolysis and oxidative phosphorylation thereby linking p53 not only to cancer, but also to other diseases such as diabetes and obesity, and to other physiological processes such as ageing (Maddocks and Vousden 2011). Thus, the repertoire of genes subject to p53 control as a master regulatory transcription factor extends across a diverse group of biological activities (Menendez et al. 2009). It has also been observed that abrogation of p53 activity by knockout or knockdown of *TP53* in human cells in vitro affects carcinogen activation (Hockley et al. 2008; Simoes et al. 2008) and drug metabolism (Goldstein et al. 2013), but as yet little is known about the mechanism of this phenomenon.

Polycyclic aromatic hydrocarbons (PAHs) are formed by the incomplete combustion of organic matter (Baird et al. 2005; IARC 2010) and are widely distributed in the environment. Several of them are highly carcinogenic, including benzo[*a*]pyrene (BaP), the most widely studied, as well as dibenz[*a,h*]anthracene (DB[*a,h*]A) and dibenzo[*a,l*]pyrene (DB[*a,l*]P) (Chang et al. 2013; Crowell et al. 2014; Lemieux et al. 2011; Siddens et al. 2012). PAHs require metabolic activation in order to exert their genotoxic effects (Baird et al. 2005; Luch and Baird 2005). Metabolic activation is catalysed predominantly by cytochrome P450-dependent monooxygenases (CYPs) resulting in highly reactive diol-epoxides capable of forming covalent DNA adducts that can lead to mutations through errors in DNA replication (Phillips 2005).

In previous studies, we observed that many of the gene expression changes induced by BaP in human cells in vitro were in genes linked to pathways of p53 function (Hockley et al. 2006, 2008). Furthermore, we found that DNA adduct formation by BaP was significantly diminished in cells in which *TP53* was knocked out or silenced (by siRNA inhibition) relative to cells with normal p53 function (Hockley et al. 2008).

In order to evaluate the impact of the cellular *TP53* status on the metabolic activation of a variety of different PAHs, a panel of isogenic human HCT116 cell lines that differ only with respect to their endogenous *TP53* status, expressing either wild-type (WT) p53 [*TP53(+/+)*], heterozygous p53 [*TP53(*+*/−)*] or mutant p53 [*TP53(R248W/+)* or *TP53(R248W/−)*], or with a complete knockout of p53 [*TP53(−/−)*] (Sur et al. 2009) was treated with BaP, DB[*a,h*]A) or DB[*a,l*]P. PAH-DNA adduct formation was determined by ^32^P-postlabelling. Expression of xenobiotic metabolising enzymes (XMEs) involved in PAH metabolism (e.g. CYP1A1) was determined by Western blotting and qRT-PCR. Cells were also treated with the reactive PAH-diol-epoxides in order to bypass the need for metabolic activation. We investigated whether p53 impacts on the regulation of XME expression such as CYP1A1 via the aryl hydrocarbon receptor (AHR) or whether the expression of XMEs such as CYP1A1 may be transcriptionally regulated by direct binding of p53 to the regulatory regions of the XME genes (Goldstein et al. 2013).

## Materials and methods

### Carcinogens

Benzo[*a*]pyrene (BaP; CAS no. 50-32-8; purity ≥96 %) was obtained from Sigma-Aldrich and (±)-*anti*-benzo[*a*]pyrene-*trans*-7,8-dihydrodiol-9,10-epoxide (BPDE) was synthesized as reported previously (Hockley et al. 2008). Dibenz[*a,h*]anthracene (DB[*a,h*]A; CAS no. 53-70-3; purity ≥99.9 %), dibenzo[*a,l*]pyrene (DB[*a,l*]P; CAS no. 191-30-0; purity ≥99.9 %), (±)-*anti*-3,4-dihydroxy-1,2-epoxy-1,2,3,4-tetrahydro-DB[*a,h*]A (DB[*a,h*]ADE) and (±)-*anti*-11,12-dihydroxy-13,14-epoxy-11,12,13,14-tetrahydro-DB[*a,l*]P (DB[*a,l*]PDE) were synthesized at the Biochemical Institute for Environmental Carcinogens, Prof. Dr. Gernot Grimmer-Foundation, Germany, according to the literature methods (Platt and Oesch 1981; Lee and Harvey 1980; Karle et al. 1977; Luch et al. 1994). The BaP metabolites (±)-*trans*-7,8-dihydroxy-7,8-dihydrobenzo[*a*]pyrene (BaP-*t*-7,8-dihydrodiol) and (±)-*r*-7,*t*-8,*t*-9,*c*-10-tetrahydroxy-7,8,9,10-tetrahydrobenzo[*a*]pyrene (BaP-tetrol-I-1) were also synthesized at the Biochemical Institute for Environmental Carcinogens using earlier published methods (Platt and Oesch 1983; Yagi et al. 1977). Mass spectrometry data and high field ^1^H-NMR spectra (400 MHz) were in essential agreement to those published previously.

### Cell culture and chemical treatment

Through targeted homologous recombination, a panel of isogenic HCT116 human colorectal carcinoma cell lines has been developed that differ only with respect to their endogenous *TP53* status. Cells expressing either WT p53 [i.e. *TP53(+/+)*], heterozygous p53 [i.e. *TP53(*+*/−)*], mutant p53 [i.e. *TP53(R248W/+)* or *TP53*(*R248W/−)*] or with a complete knockout of p53 [i.e. *TP53(−/−)*] (Sur et al. 2009) were kindly provided by Prof. Bert Vogelstein, John Hopkins University School of Medicine, Baltimore, MD. The R248 W mutation is found in some patients with the Li–Fraumeni syndrome and leads to substitution of arginine for tryptophan, which results in modulated DNA binding capacity of the corresponding p53 protein product.

HCT116 cells were grown as adherent monolayers in complete growth medium: Dulbecco’s modified Eagle’s medium (#21885-025, Invitrogen) with 10 % foetal bovine serum (#10106, Invitrogen), supplemented with 100 units/mL penicillin–streptomycin. Cells were cultured at 37 °C in 5 % CO_2_ and passaged before the cells surpassed 80 % confluence. For treatment, cells were seeded at 3 × 10^4^ cells/cm^2^ in flasks, grown for 48 h and subsequently treated with the test compounds or solvent dimethyl sulfoxide (DMSO) as a control for up to 48 h. The DMSO concentration was always kept at ≤0.5 % of the total culture medium volume. Based on previous experiments (Hockley et al. 2008), cells were treated with 2.5 µM BaP, DB[*a,h*]A or DB[*a,l*]P. Incubations with PAH-diol-epoxides were as follows: 0.5 µM BPDE, 0.5 µM DB[*a,h*]ADE or 0.0025 µM DB[*a,l*]PDE. Cells were harvested by trypsinization and washed with phosphate-buffered saline (PBS).

In experiments using nutlin-3a (Cayman Chemicals; 18585), cells were pretreated with 5 µM nutlin-3a for 6 h prior to exposure to BaP for 24 h. Nutlin-3a remained for the whole incubation period.

### Cell viability and DNA adduct analysis

Cell viability (% control) was measured using the CASY Model TT Electronic Cell Analyser (Innovatis AG, Germany). DNA was isolated from PAH-treated cells using a standard phenol/chloroform extraction method. The nuclease P1 digestion enrichment version of the thin-layer chromatography (TLC) ^32^P-postlabelling assay was used to measure DNA adduct formation. The procedure was essentially carried out as described previously (Arlt et al. 2008; Malik et al. 2013; Phillips and Arlt 2007; Siddens et al. 2012). After chromatography, TLC sheets were scanned using a Packard Instant Imager (Dowers Grove, IL, USA) and DNA adduct levels (RAL, relative adduct labelling) were calculated from the adduct cpm, the specific activity of [γ-^32^P]ATP and the amount of DNA (pmol of DNA-P) used.

### Western blot analysis

After treatment cells were lysed [62.5 mM Tris (pH 6.8), 1 mM EDTA (pH 8.0), 2 % sodium dodecyl sulphate (SDS), 10 % glycerol], cells were sonicated and centrifuged for 5 min at 5,000 rpm. Subsequently, protein concentration of the supernatant was determined using the BCA Protein Assay (Pierce, Thermo Scientific, UK) according to the manufacturer’s instructions. β-Mercaptoethanol (0.1 % final; in a solution containing the loading dye bromophenol blue) was added to reduce disulphide bonds and lysates were denatured at 95 °C for 5 min. Equal amounts of protein (10–20 µg) were separated by SDS–polyacrylamide gel electrophoresis (SDS-PAGE) using 4–12 % Bis–Tris gradient or 10 % Bis–Tris gels, and Western blotted as previously reported (Kucab et al. 2012). After blocking in 3 % nonfat milk (dissolved in PBS with 0.2 % Tween-20), blots were incubated overnight at 4 °C with primary antibodies or anti-serum diluted in blocking solution. The following primary antibodies and dilutions were used: anti-p53 1:2,000 (Ab-6, Calbiochem), anti-p21 (CDKN1A) 1:2,000 (556431, BD Pharmingen), anti-NQO1 1:10,000 (ab34173, Abcam) and anti-AHR 1:1,000 (ab2770, Abcam). Anti-CYP1A1 raised in rabbits against purified human recombinant CYP1A1 was a generous gift from Prof. F. Peter Guengerich (Vanderbilt University, USA) and was diluted 1:4000. The antibody to detect β-actin 1:20,000 (ab6276, Abcam) was used as loading control. The secondary horseradish peroxidase-linked antibodies were as follows: anti-mouse (#170-5047; 1:10,000) and anti-rabbit (#170-5046; 1:10,000) from BioRad.

### Measurement of nucleotide excision repair (NER) capacity

To assess NER capacity in HCT116 cells, a modified comet assay was applied (Langie et al. 2006). This assay measures the ability of NER-related enzymes that are present in the cell extract to incise substrate DNA containing BPDE-DNA adducts. The substrate nucleoids were prepared from untreated HCT116 *TP53(+/+)* cells as reported (Langie et al. 2006). They were then exposed to BPDE (1 µM) or vehicle control (DMSO, 0.5 % in PBS) for 30 min. Preparation of the protein extracts of the HCT116 *TP53(+/+)*, *TP53(*+*/−)*, *TP53(−/−)*, *TP53(R248W/+)* and *TP53(R248W/−)* cells, ex vivo repair incubation and electrophoresis were performed according to the published protocol (Langie et al. 2006). Dried slides were stained with ethidium bromide (10 µg/mL), and comets were analysed using a Leica fluorescence microscope (Leica DMLB 020-519-010 LB30T). DNA damage was scored using the Comet IV capture system (version 4.11; Perceptive Instruments, UK). Fifty nucleoids were assessed per slide and each sample was analysed in duplicate. All samples were measured blindly. The results from both replicates were combined for further analysis. The tail intensity (% tail DNA), defined as the percentage of DNA migrated from the head of the comet into the tail, was used as an indicative measure of the repair capacity of the cell extracts. After subtracting background levels from all data, the final DNA repair capacity was calculated as previously reported (Langie et al. 2006).

### High-performance liquid chromatography (HPLC) analysis of BaP metabolites

For the analysis of BaP metabolites, culture medium (2 mL) from exposed cells was collected and stored at −20 °C until further processing. These samples were analysed at the National Institute of Occupational Health, Norway, as described (Dendele et al. 2014). Briefly, the medium was diluted to 10 mL with water, applied to a preconditioned (5 mL methanol and 10 mL water) Sep-Pak C18 cartridge (Millipore Corporation, Milford, MA, USA), followed by a wash with water (10 mL), and eluted with 100 % methanol (5 mL). The methanol eluate was evaporated to dryness at 45 °C under a nitrogen stream and suspended in 100 μL of 100 % methanol. HPLC separation of BaP metabolites was performed on a Nova-Pak C18 3.9 × 150 mm^2^ column (Waters, Milford, MA, USA) with a Waters 625 LC System, equipped with a LC 240 fluorescence detector (Perkin-Elmer, Beaconsfield, UK) (Dendele et al. 2014). The BaP metabolites were separated with a linear gradient of 30–100 % methanol in water for 40 min. For the quantitative determination of BaP metabolites, the following fluorescence conditions were used: 0 min, excitation 380 nm, emission 431 nm; 0.5 min, excitation 341 nm, emission 381 nm; 20 min, excitation 253 nm, emission 410 nm; and 27 min, excitation 380 nm, emission 431 nm. The concentrations of BaP metabolite standards dissolved in ethanol were determined by UV absorbance using extinction coefficients supplied by the NIH Chemical Carcinogen Repository (Midwest Research Institute, Kansas City, MO, USA).

In experiments using nutlin-3a, selected BaP metabolites were analysed at King’s College London. Briefly, 5 mL media was removed and extracted twice with 1 mL of ethyl acetate. Extracts were evaporated and taken up in 200 µL methanol, of which 20-µL aliquots were injected. HPLC analysis was performed using a HPLC Agilent 1100 System (Agilent Technologies) with a SunFireTM C18 reverse phase column (250 × 4.6 mm, 5 µm; Waters) using a methanol/water gradient at a flow rate of 0.2 mL/min. A linear gradient of 20–100 % methanol over 10 min was followed by isocratic elution for 20 min. This was succeeded by a linear gradient of 100–20 % methanol for 10 min, and then isocratic elution of 20 % methanol for 5 min. Total run time was 60 min. The metabolites were analysed by fluorescence detection (excitation wavelength 375 nm, emission wavelength 450 nm). The two BaP metabolites analysed, BaP-*t*-7,8-dihydrodiol and BaP-tetrol-I-1, were identified using authentic standards.

### Cell cycle

After treatment with 2.5 μM BaP for 24 and 48 h, HCT116 cells were harvested and fixed in ice cold 70 % ethanol. For staining with propidium iodide (Invitrogen, UK), cells were pelleted, washed with PBS and resuspended in staining buffer containing 40 μg/mL propidium iodide, 100 μg/mL RNase (Qiagen, UK) in PBS. Samples were incubated at 37 °C for 1 h in the dark and subsequently stored at 4 °C overnight before analysis. The DNA content of 10,000 cells per sample was analysed using a BD FACSCanto II (BD Biosciences, UK) at 488 nm.

### Gene expression analysis

HCT116 cells were treated with 2.5 μM BaP for 24 h. RNA was isolated using RNeasy Mini kit (Qiagen, UK) and reverse-transcribed into cDNA using the SuperScript III reverse transcriptase kit (Invitrogen, UK). Relative quantitation of *CYP1A1* and *CYP1B1* mRNA expression was performed using fluorescent qRT-PCR with the ABI PRISM 7500HT Fast Sequence Detection System (Applied Biosystems, UK) as described previously (Hockley et al. 2006, 2008). *CYP1A1* and *CYP1B1* expression was detected using TaqMan^®^ gene expression primers and probes (*CYP1A1*-Hs00153120_m1 and *CYP1B1*-Hs00164383_m1). Relative gene expression was calculated using the comparative threshold cycle (*C*_T_) method.

### Chromatin immunoprecipitation (ChIP)

HCT116 *TP53(+/+)* and *TP53(−/−)* cells were treated with 2.5 μM BaP for 24 h. In order to specifically induce p53 and its target p21, cells were also treated with 10 μM nutlin-3a. Cells underwent cross-linking (in 0.75 % formaldehyde for 10 min at room temperature) followed by quenching with glycine (in 0.125 M glycine for 5 min at room temperature). Cells were rinsed twice with 10 mL cold PBS, scraped and centrifuged (5 min, 1,000×*g*). Subsequently, cells were lysed (50 mM HEPES–KOH pH 7.5, 140 mM sodium chloride, 1 mM EDTA, 0.1 % SDS, 1 % Triton X-100, 0.1 % sodium deoxycholate, and 1 % protease inhibitors), sonicated to an average DNA fragment length of 500 base pairs (6 × 15 s) and then centrifuged (15 min, 8 000×*g*, 4 °C). The chromatin solution was precleared by adding protein A beads (sc-2001, Santa Cruz Biotechnology) that were pre-absorbed with BSA and salmon sperm DNA for 1 h at 4 °C. Immunoprecipitation of chromatin was performed with 5 μL of polyclonal α-p53 antibody (CM1, Cambridge Bioscience) or with nonrelevant rabbit anti-IgG (Abcam, ab46540) overnight at 4 °C. Immunoprecipitates were washed three times with washing buffer (20 mM Tris–HCl pH 8.0, 500 mM sodium chloride, 1 % Triton X-100, 2 mM EDTA pH 8.0, 0.1 % SDS, and 1 % protease inhibitors). Samples were treated with 2 µL RNase A (0.5 mg/mL) for 30 min (37 °C), followed by 30 µL proteinase K (20 mg/mL) for 2 h, (37 °C). DNA was isolated by phenol/chloroform extraction. qRT-PCR was performed as described above with each sample containing 5 µL of immunoprecipitated DNA; values were normalised for 1 % input values. Five independent experiments were performed.

ChIP primer sequences for the *CDKN1A* promoter (5′ p53 response element) were adapted from previous studies (Kaeser and Iggo 2002; Laptenko et al. 2011). ChIP primer sequences for the *CYP1A1* promoter (5′ p53 response element) were adapted from Rockefeller University (http://linkage.rockefeller.edu/p53; 16 October 2012) and verified by bioinformatic analysis. Primers are in italics, TaqMan probes are in bold, and p53-binding sites (*CDKN1A*, *CYP1A1*) are underlined:

- *CDKN1A* promoter:
- *CTGGACTGGGCACTCTTGTC*CCCCAGGCTGAGCCTCCCTCCATCCCTATGCTGCCTGCTTCCCAGGAACATGCTTGGGCAGCAGGCTGTGGCTCTGATTGGCTTTCTGGCCGTCAGGAACATGTCCCAACATGTTGAGCTCTGGCATAGAAGA**GGCTGGTGGCTATTTTGTCC**TTGGGCTGCC*TGTTTTCAGGTGAGGAAGGG*
- *CYP1A1* promoter:
- *AGCTTCAGGCTACTGCAAGG*AACAACCAAGCTGAAGTCAGCTGCGGCAACCTGCTTTGTGCAGCGGCGGCCGG**GGGGATGAGAAATTTGGTGC**TCAATCATTCTTGTAGTGATTTATATTTTCTACTTAAACTATAACTTGCATTTATGCTATATACATAAACAAGCCATGAAAGCATGCTA *CGTGAAAGAAGCCAGACACA*

## Results

### Cell viability after PAH exposure

In the previous work, treatment with 2.5 μM BaP and 0.5 μM BPDE was found to induce DNA adduct formation in HCT116 *TP53(+/+)* cells within a linear dose–response range (Hockley et al. 2008). The optimal concentrations of DB[*a,h*]A and DB[*a,l*]P were determined in initial experiments to be similar to that of BaP (data not shown). Thus, HCT116 cells were treated with 2.5 μM BaP, DB[*a,h*]A or DB[*a,l*]P, which allowed direct comparison of the potency of each PAH to induce DNA adduct formation. Whereas DB[*a,h*]ADE could be tested at the same concentration as BPDE, the high cytotoxicity of DB[*a,l*]PDE meant that the selected concentration for treatment was 200-fold lower, at 0.0025 μM. Under these treatment conditions, no impact on cell viability (>80 % relative to controls) was observed in any of the isogenic HCT116 cell lines after PAH exposure for 24 and 48 h (Supporting Figure 2), which was in line with previous findings (Hockley et al. 2008).

### Expression of DNA damage response proteins and cell cycle alteration after PAH exposure

**Fig. 1:**
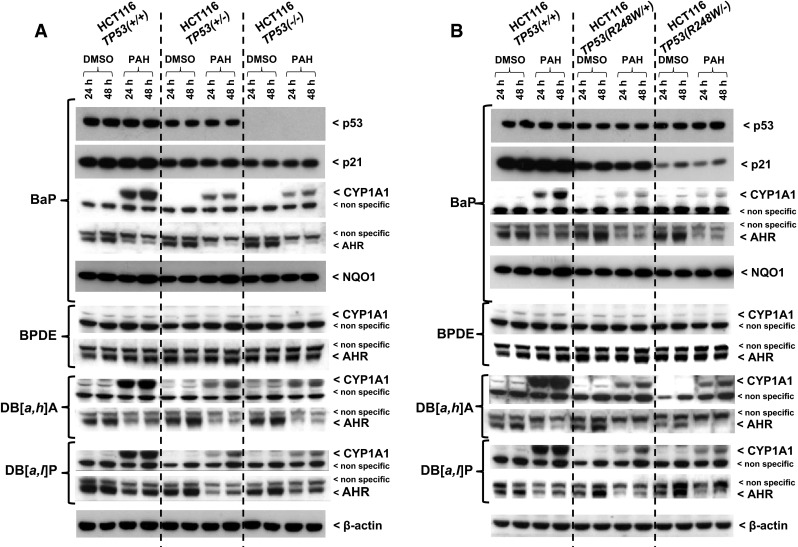
Western blot analysis of p53, p21 (CDKN1A), CYP1A1, AHR and NQO1 protein expression in isogenic HCT116 cells after exposure to various PAHs or BPDE. Cells were exposed to 2.5 μM BaP, 0.5 μM BPDE, 2.5 μM DB[*a,h*]A or 2.5 μM DB[*a,l*]P and harvested after the times indicated. **a** HCT116 *TP53(+/+)* cells compared to *TP53(*+*/−)* and *TP53(−/−)* cells. **b** HCT116 *TP53(+/+)* cells compared to *TP53(R248W/+)* and *TP53(R248W/−)* cells. β-Actin protein expression was used as a loading control. Representative images of the Western blotting are shown; at least duplicate analysis was performed from independent experiments

Expression of DNA damage response proteins p53 and p21 was assessed by Western blot analysis after BaP exposure for 24 and 48 h (Fig. 1). Each cell line displayed a time-independent and treatment-independent level of p53 expression, except for *TP53(−/−)* cells which did not express p53 as expected. Equal expression levels were detectable in *TP53(+/+)*, *TP53*(*R248W/+)* and *TP53*(*R248W/−)* cells (Fig. 1b), whereas p53 levels decreased in *TP53(*+*/−)* relative to *TP53(+/+)* cells (Fig. 1a). The p53 target p21 was expressed in all cell lines, but no induction was found after BaP treatment compared with control (untreated) samples (Fig. 1). The highest p21 levels were observed in *TP53(+/+)* cells. Expression levels were reduced in *TP53(*+*/−)* cells and further reduced in *TP53(−/−)* cells (Fig. 1a). *TP53(R248W/+)* cells showed similar levels of p21 expression to *TP53(−/−)* cells, whereas hardly any p21 expression was seen in *TP53(R248W/−)* cells (Fig. 1b).

As shown in Supporting Figure 3A, cell cycle parameters as measured by flow cytometry were modestly altered after 24 h in *TP53(R248W/+)* and *TP53(R248W/−)* relative to *TP53(+/+)* cells. After 48 h, BaP treatment had a marginal effect on cell cycle in *TP53(+/+)* cells relative to controls (Supporting Figure 3B), consistent with previous results (Hockley et al. 2008).

### Formation of PAH-DNA adducts

**Fig. 2:**
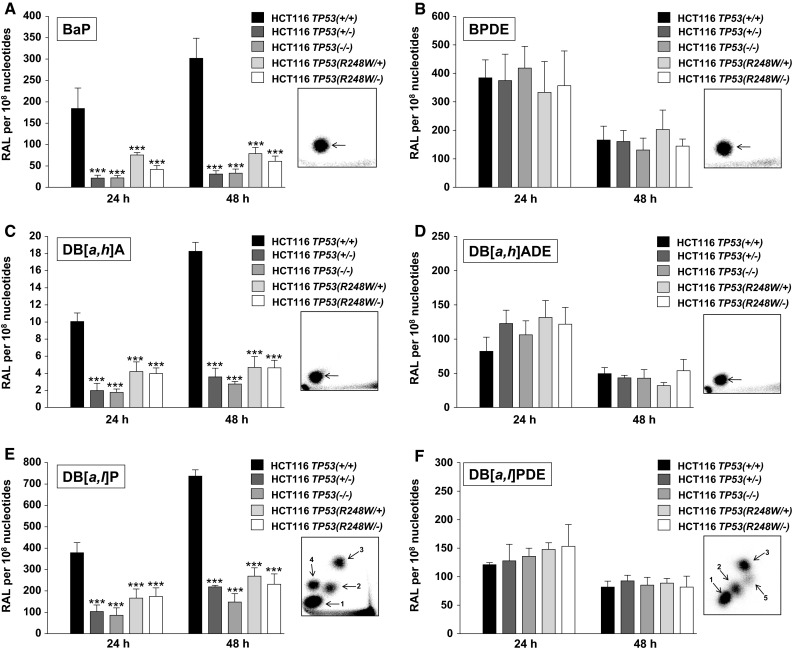
DNA adduct levels (RAL, relative adduct labelling) detected by ^32^P-postlabelling in isogenic HCT116 cells after exposure to various PAHs and their corresponding diol-epoxides. Cells were exposed to **a** 2.5 μM BaP, **b** 0.5 μM BPDE, **c** 2.5 μM DB[*a,h*]A, **d** 0.5 μM DB[*a,h*]ADE, **e** 2.5 μM DB[*a,l*]P or **f** 0.0025 μM DB[*a,l*]PDE and harvested after the times indicated. The values are the mean ± range of duplicate cell incubations; each DNA sample was analysed in duplicate in separate experiments. Statistical analysis was performed by one-way ANOVA followed by the Tukey post hoc test [***p < 0.005, different from HCT116 *TP53(+/+)* cells]. *Insets* Autoradiographic profiles of DNA adducts formed in HCT116 cells after exposure; the origins, at the *bottom left-hand corners*, were cut off before exposure

PAH-DNA adduct formation after 24 and 48 h was determined by the ^32^P-postlabelling method (Fig. 2). After BaP and BPDE treatment (Fig. 2a, b), the DNA adduct pattern on TLC was qualitatively similar in all isogenic HCT116 cells, consisting of one single adduct spot, previously identified as 10-(deoxyguanosin-*N* ^2^-yl)-7,8,9-trihydroxy-7,8,9,10-tetrahydrobenzo[*a*]pyrene (dG-*N* ^2^-BPDE) (Arlt et al. 2008). DB[*a,h*]A treatment also resulted in the formation of one major adduct spot which corresponded to the adduct spot obtained after treatment with DB[*a,h*]ADE (Fig. 2c, d). Exposure to DB[*a,l*]P induced a pattern consisting of four major adduct spots (Fig. 2e), of which three were found after treatment with DB[*a,l*]PDE (i.e. spots 1–3; Fig. 2f). No DNA adducts were detected in controls (data not shown).

Quantitative analysis obtained by ^32^P-postlabelling revealed a time-dependent increase in DNA adduct formation from 24 to 48 h after treatment with the parent PAH (Fig. 2a, c, e). In *TP53(+/+)* cells, adduct levels were ~twofold higher and up to 1.5-fold higher for the other cell lines at 48 h than at 24 h. Amongst the PAHs, the genotoxic potencies increased in the order DB[*a,h*]A ≪ BaP < DB[*a,l*]P. Strikingly, PAH-induced DNA adduct levels were up to eightfold lower in *TP53(−/−)* than in *TP53(+/+)* cells; the difference was ~eightfold for BaP, ~sixfold for DB[*a,h*]A and ~fourfold for DB[*a,l*]P. Interestingly, adduct levels in *TP53(*+*/−)* cells were almost the same as those found in *TP53(−/−)* cells. It is also noteworthy that adduct levels in both mutant *TP53* cell lines, *TP53(R248W/+)* and *TP53(R248W/−)*, were manifoldly lower compared with *TP53(+/+)* cells, but generally ~1.5 times higher than in *TP53(−/−)* cells.

In contrast, similar adduct levels in *TP53(+/+)* cells relative to all other cell lines were observed after treatment with BPDE (Fig. 2b). In line with this observation, adduct levels were also similar after DB[*a,h*]ADE or DB[*a,l*]PDE exposure in all cell lines (Fig. 2d, f). For all three PAH-diol-epoxides, DNA adduct formation was lower after 48 h relative to 24-h treatment. It is also noteworthy that adduct levels after shorter diol-epoxide exposure, namely 2 h, were similar in all cell lines (data not shown). More importantly, as PAH-diol-epoxides do not require metabolic activation to bind to DNA, these findings suggest that the differences in DNA adduct formation observed with the parent PAHs are the consequence of the different capacities of the cells to metabolically activate the PAHs. In other words, these results suggest that the cellular *TP53* status impacts on the metabolic bioactivation of the parent PAHs.

### CYP1A1 protein expression after PAH exposure

Bioactivation of many PAHs including BaP, DB[*a,h*]A and DB[*a,l*]P is catalysed by CYP1A1 (Baird et al. 2005; Luch and Baird 2005). To determine the impact of the cellular *TP53* status in HCT116 cells on the CYP1A1-mediated metabolic activation of the PAHs tested, CYP1A1 protein levels were determined by Western blotting after 24 and 48 h. As shown in Fig. 1, basal levels of CYP1A1 were low in all cell lines, but CYP1A1 was induced after PAH exposure. A striking finding was that CYP1A1 induction correlated with the PAH-induced DNA adduct levels in each cell line (compare Fig. 2). In contrast, after BPDE exposure, for example, no CYP1A1 induction was observed (Fig. 1). The lack of CYP1A1 induction after BPDE correlated with the finding that BPDE exposure of the cells resulted in similar DNA adduct levels regardless of the *TP53* status (compare Fig. 2b).

PAHs such as BaP, DB[*a,h*]A and DB[*a,l*]P can bind to and activate AHR (Hockley et al. 2007). Thus, the activated receptor is capable of modulating the expression of its target genes such as *CYP1A1* (Hockley et al. 2007). As shown in Fig. 1, AHR expression in whole cell lysates decreased in all cell lines to the same extent after PAH exposure.

### CYP1A1 and CYP1B1 mRNA expression after BaP treatment

**Fig. 3:**
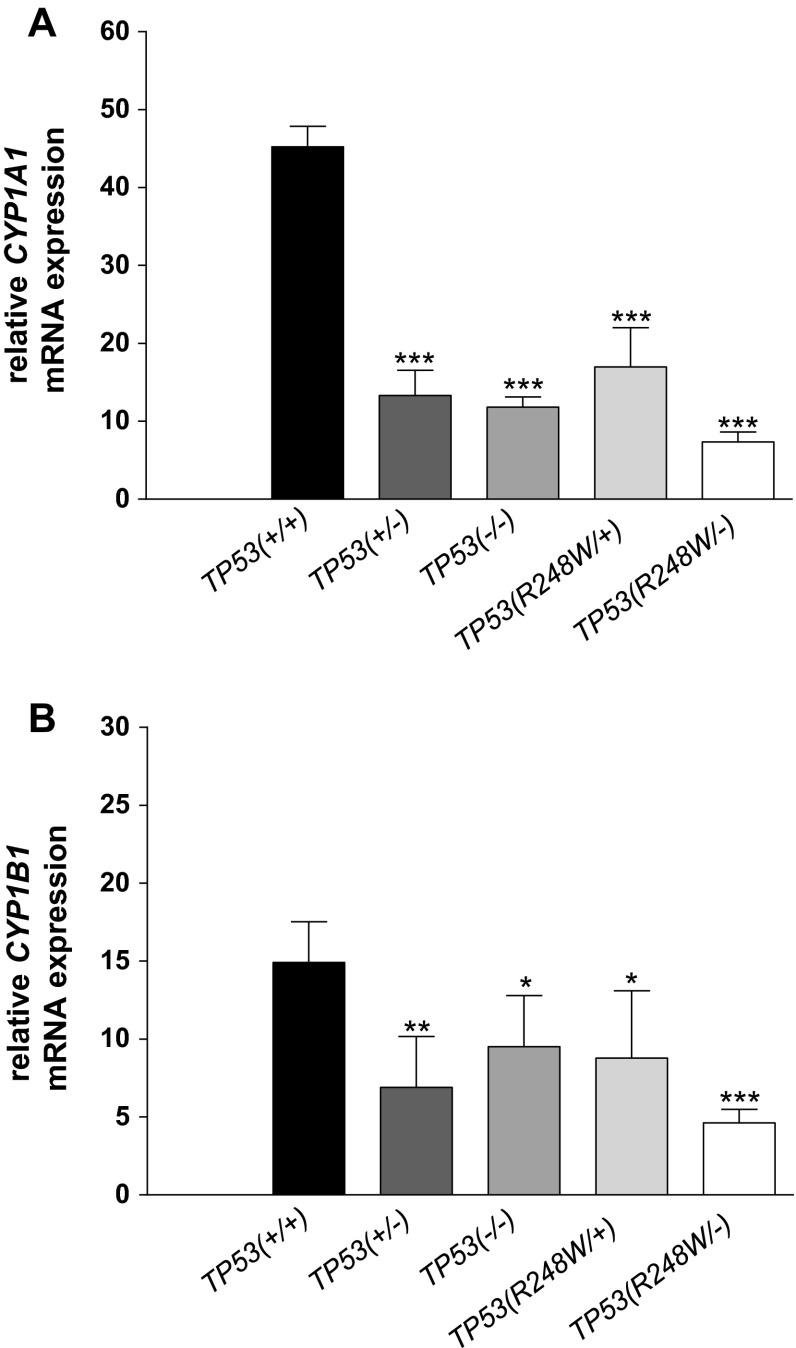
Gene expression of **a** *CYP1A1* and **b** *CYP1B1* in isogenic HCT116 cells after exposure to 2.5 μM BaP for 24 h. Total RNA was extracted and the mRNA levels of the indicated genes were analysed by qRT-PCR. Values represent the mean ± SD of three incubations; each sample was determined by three separate analyses. Statistical analysis was performed by one-way ANOVA followed by the Tukey post hoc test [* *p* < 0.05; ** *p* < 0.01; *** *p* < 0.005, different from HCT116 *TP53(+/+)* cells]

RT-PCR is a sensitive and specific measure of gene expression (Hamouchene et al. 2011; Hockley et al. 2006) and was used to evaluate *CYP1A1* and *CYP1B1* expression changes in HCT116 cells treated with BaP for 24 h. Similar to the results obtained by Western blotting, *CYP1A1* mRNA induction after BaP treatment was lower in all cells relative to *TP53(+/+)* cells (Fig. 3a) and correlated with BaP-DNA adduct formation in each cell line (compare Fig. 1a). Small alterations in *CYP1B1* mRNA induction were found in some cell lines (Fig. 3b); again *CYP1B1* expression was lower relative to *TP53(+/+)* cells in all other cell lines. However, the fold differences appeared to be subtle, suggesting that any change in *CYP1B1* expression may not significantly influence the differences in BaP-DNA adduct levels observed in these cell lines.

### NQO1 protein expression after BaP treatment

As BaP-derivatives can also be metabolised by NAD(P)H:quinone oxidoreductase (NQO1) (Luch and Baird 2005), we also determined NQO1 protein expression in HCT116 cells after BaP exposure. It appears that a marginal induction of NQO1 protein levels was observed upon BaP treatment after 48 h (Fig. 1). However, this induction was very small and independent of the cellular *TP53* status.

### Impact of cellular *TP53* status on BaP metabolism

In order to obtain further insights into the effects of *TP53* status on BaP metabolism, BaP metabolites were analysed in the cell culture medium by HPLC analysis. A scheme showing the structures of BaP metabolites analysed is given in Supplementary Figure 4. As shown in Table 1, the levels of all BaP metabolites analysed in the *TP53(+/+)* cells were significantly higher than in all other cultures. For example, the level of BaP-*t*-7,8-dihydrodiol, a precursor of BPDE, was ~fivefold higher in *TP53(+/+)* than in *TP53(−/−)* cell cultures. Overall, the levels of BaP metabolites formed by each cell line corresponded to the BaP-DNA adduct levels observed in these cells.

**Table 1:** HPLC analysis of BaP metabolites in HCT116 cell culture medium

BaP/BaP metabolite^a^	pmol BaP/BaP metabolite (mean ± SD [*n* = 4])^b^				
	HCT116 *TP53(+/+)*	HCT116 *TP53(*+*/−)*	HCT116 *TP53(−/−)*	HCT116 *TP53(R248W/+)*	HCT116 *TP53(R248W/−)*
BaP-*t*-7,8-dihydrodiol	63.2 ± 4.7	17.3 ± 0.8***	13.3 ± 0.7***	41.6 ± 3.8***	34.6 ± 2.7***
BaP-*c*-7,8-dihydrodiol	0.6 ± 0.1	0.2 ± 0.1*	0.4 ± 0.3	0.5 ± 0.1	0.4 ± 0.1^*^
BaP-tetrol-I-1^c^	16.4 ± 1.5	0.7 ± 0.1***	0.6 ± 0.1***	2.8 ± 0.2***	1.6 ± 0.2***
BaP-tetrol-I-2^c^	1.6 ± 0.1	0.4 ± 0.01***	0.3 ± 0.03***	0.8 ± 0.1***	0.6 ± 0.1***
BaP-tetrol-II-1^c^	2.0 ± 0.1	0.1 ± 0.1***	0.1 ± 0.1***	0.4 ± 0.02***	0.2 ± 0.1***
BaP-tetrol-II-2^c^	6.1 ± 0.2	1.1 ± 0.1***	1.1 ± 0.1***	2.8 ± 0.1***	2.1 ± 0.2***
BaP-4,5-dihydrodiol	3.1 ± 0.3	1.1 ± 0.2***	0.9 ± 0.1***	1.8 ± 0.2***	1.6 ± 0.4***
BaP-9,10-dihydrodiol	119.1 ± 4.7	21.6 ± 1.2***	21.2 ± 2.0***	52.7 ± 1.3***	37.5 ± 4.0***
BaP-3-ol	ND^d^	0.1 ± 0.1	0.1 ± 0.1	ND	ND
BaP-9-ol	0.6 ± 0.1	0.4 ± 0.1***	0.3 ± 0.01***	0.2 ± 0.02***	0.1 ± 0.01***
BaP	319.8 ± 35.7	483.3 ± 37.8***	403.8 ± 28.2	447.8 ± 47.8*	579.5 ± 89.6***

^a^Structures of the BaP metabolites detected by HPLC are shown in Supplementary Figure 4

^b^Statistical analysis was performed by one-way ANOVA followed by Tukey post hoc test [* *p* < 0.05; *** *p* < 0.005, different from HCT116 *TP53(+/+)* cells]

^c^BaP-tetrol-I-1: BaP-*r*-7,*t*-8,*t*-9,*c*-10-tetrahydrotetrol; BaP-tetrol-I-2: BaP-*r*-7,*t*-8,*t*-9,*t*-10-tetrahydrotetrol; BaP-tetrol-II-1: BaP-*r*-7,*t*-8,*c*-9,*t*-10-tetrahydrotetrol; BaP-tetrol-II-2: BaP-*r*-7,*t*-8,*c*-9,*c*-10-tetrahydrotetrol

^d^ND not detected

### Impact of p53 induction on BaP metabolism and CYP1A1 expression

**Fig. 4:**
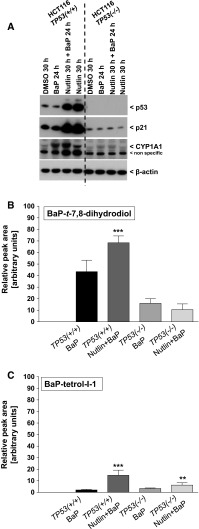
**a** Western blot analysis of p53 and CYP1A1 protein expression in HCT116 *TP53(+/+)* and *TP53(−/−)* cells after pre-treatment with nutlin-3a (5 μM) for 6 + 24 h and co-exposure with 2.5 μM BaP for 24 h. Representative images of the Western blotting are shown and duplicate analysis was performed from independent experiments. HPLC analysis of BaP-*t*-7,8-dihydrodiol (**b**) and BaP-tetrol-I-1 (**c**) in the culture medium of HCT116 *TP53(+/+)* and *TP53(−/−)* cells after pre-treatment with nutlin-3a (5 μM) for 6 + 24 h and co-exposure with 2.5 μM BaP for 24 h. Values represent the mean ± SD of at least three separate incubations. Statistical analysis was performed by one-way ANOVA followed by the Tukey post hoc test [**p < 0.01; ***p < 0.005, different from BaP treated only]

Nutlin-3a, an inhibitor of MDM2, was used to induce p53 in *TP53(+/+)* cells. Using Western blot analysis, it was found that p53 was induced upon nutlin-3a pre-treatment which subsequently led to an increase in CYP1A1 levels in cells co-exposed to BaP for 24 h (Fig. 4a). CYP1A1 protein levels were slightly induced in cells treated solely with nutlin-3a for up to 30 h. Similar experiments carried out in *TP53(−/−)* cells showed a marginal induction of CYP1A1 levels after treatment with BaP alone, but pre-treatment with nutlin-3a had no effect on CYP1A1 expression levels (Fig. 4a).

The p53/nutlin-3a-mediated induction of CYP1A1 in *TP53(+/+)* cells after BaP exposure also altered the formation of BaP metabolites. For these experiments, the formation of BaP-*t*-7,8-dihydrodiol and BaP-tetrol-II-1 was determined in the cell culture medium using HPLC analysis. These metabolites were chosen as they are, in the first case, precursors of BPDE and, in the second case, one of the hydrolysis products of BPDE. As shown in Fig. 4b, c, the levels of both BaP metabolites increased in *TP53(+/+)* cells and co-exposed to nutlin-3a and BaP relative to cells treated with BaP alone, indicating that p53 induction by nutlin-3a increases CYP1A1-mediated BaP metabolism. As seen before, the levels of both BaP metabolites were lower in *TP53(−/−)* compared with *TP53(+/+)* cells (Fig. 4b, c; compare Table 1). In *TP53(−/−)* cells, no change was observed for BaP-*t*-7,8-dihydrodiol, whereas BaP-tetrol-I-1 was significantly higher in *TP53(−/−)* cells treated with nutlin-3a and BaP relative to cells treated with BaP alone, but the change was small.

### Binding of p53 to the DNA-regulatory elements of p53 (p53REs) in the promoter region of CYP1A1 after BaP exposure

**Fig. 5:**
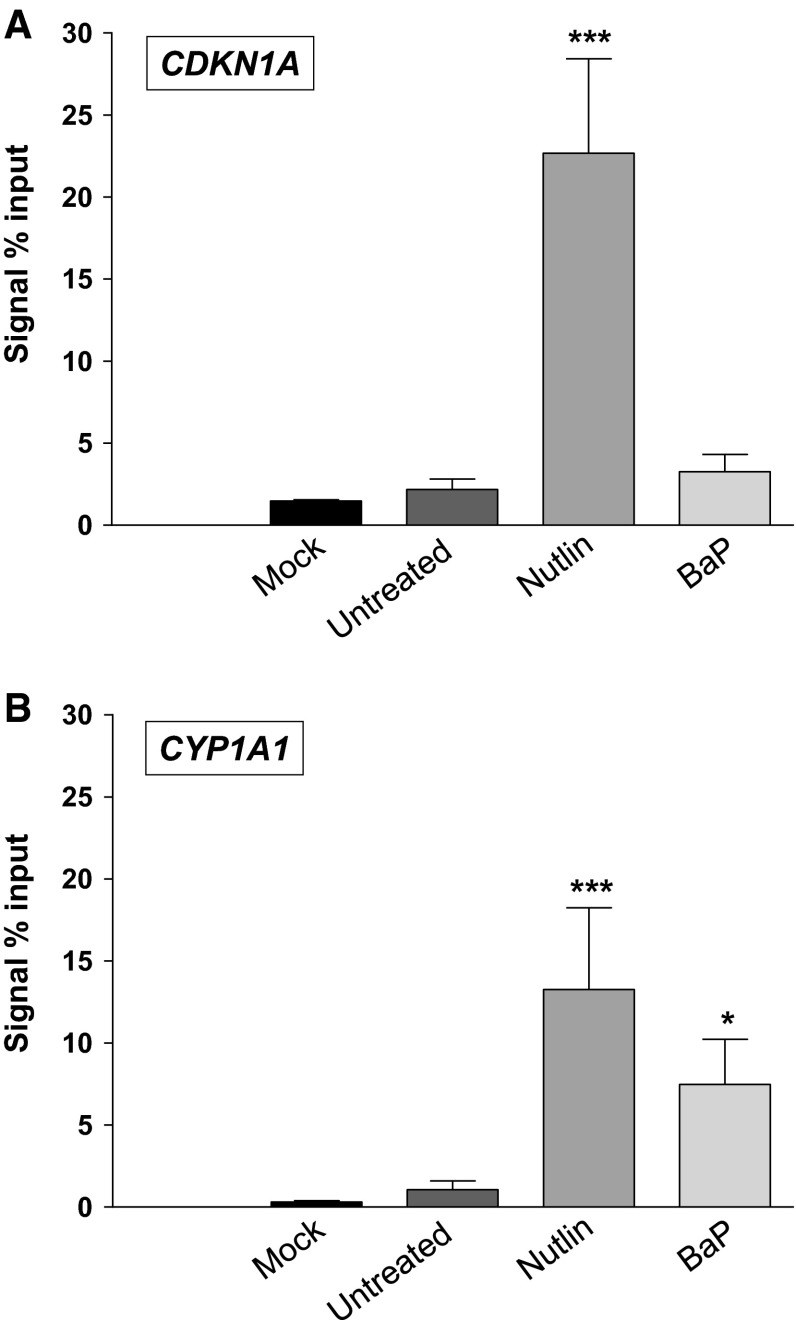
Binding of p53 to the DNA-regulatory elements (p53RE) of **a** CDKN1A and **b** CYP1A1 in HCT116 *TP53(+/+)* after exposure to 2.5 μM BaP for 24 h. HCT116 *TP53(+/+)* were also treated with nutlin-3a (10 μM) for 6 + 24 h. After chromatin immunoprecipitation (ChIP) with anti-p53 antibody, the immunoprecipitated DNA was subjected to qRT-PCR using primers amplifying the indicated p53RE regions as outlined in the “Materials and methods”. Values represent the mean ± SD of at least three separate experiments. Statistical analysis was performed by one-way ANOVA followed by the Tukey post hoc test [*p < 0.05; ***p < 0.005, different from control (untreated)]

After demonstrating that alterations in the cellular p53 levels can impact on the CYP1A1-mediated metabolism of BaP and subsequent formation of BaP-DNA adducts, we wanted to explore the potential mechanism behind this phenomenon. It is well known that p53 induces gene expression of its target genes by binding to p53REs and thereby inducing their transcription (Menendez et al. 2009). Recent studies have indicated that p53 can induce gene expression of *CYP3A4* by binding to p53REs (Goldstein et al. 2013). Therefore, we hypothesised that *CYP1A1* expression may also be regulated by p53. After in silico analysis of the regulatory region upstream of the *CYP1A1* gene, a putative p53RE was identified. We used ChIP to investigate whether p53 can bind to the p53RE identified in the regulatory region of *CYP1A1*. In order to validate the efficiency of the ChIP assay, we confirmed that p53 binds to a known p53RE of *CDKN1A* (p21) (Kaeser and Iggo 2002; Laptenko et al. 2011), a downstream target of p53, in *TP53(+/+)* cells after treatment with nutlin-3a (Fig. 5a). No increase in binding was observed in the BaP treated compared to the negative (mock) control or untreated *TP53(+/+)* cells. The lack of p53 binding to the p53RE of *CDKN1A* (p21) is in line with the Western blot analysis where no p21 induction was observed after BaP treatment in *TP53(+/+)* cells under the experimental conditions used (see Fig. 1).

Most importantly, as shown in Fig. 5b, p53 was able to bind to the p53RE of *CYP1A1* as indicated by the enrichment of this DNA segment in the p53-precipitated DNA, after both nutlin-3a and BaP treatments. Again, no increase in binding was observed in the negative (mock) control or in untreated *TP53(+/+)* cells. These results support a p53-dependent induction mechanism of CYP1A1, thereby promoting CYP1A1-mediated BaP metabolism and subsequent BaP-induced DNA adduct formation.

### DNA repair capacity

Nucleotide excision repair (NER) is the main DNA repair pathway for the studied PAHs, and p53-dependent pathways affecting global NER have been identified (Ford 2005; Sengupta and Harris 2005). In order to determine whether the cellular *TP53* status impacted on NER in HCT116 cells, we phenotypically assessed NER capacity. Cell extracts from HCT116 cells were examined for their ability to repair BPDE-induced DNA adducts using a modified comet assay (Langie et al. 2006). Utilising this assay, we found that all HCT116 cell lines used in the present study had the same NER capacity (Supporting Figure 5).

## Discussion

The repertoire of genes subject to p53 control extends across a diverse group of biological activities that include apoptosis, cell cycle regulation, senescence, energy metabolism, angiogenesis, cell differentiation and immune response. In this study, we show that carcinogenic PAHs can induce CYP1A1 in human cells via a p53-dependent mechanism, indicating a novel role for p53 in CYP1A1-mediated carcinogen metabolism. CYP1A1 is one of the key enzymes involved in PAH metabolism, and we found that alterations of the cellular *TP53* status as well as nutlin-3a-induced p53 activation had dramatic effects on the metabolism of a variety of PAHs, namely BaP, DB[*a,h*]A and DB[*a,l*]P.

Using a panel of isogenic colorectal HCT116 cells differing only with respect to their endogenous *TP53* status, we found that complete loss of p53 function resulted in considerably lower PAH-DNA adduct levels compared with cells having wild-type p53 (Fig. 1). This is in line with a previous observation using the HCT116 *TP53(+/+)* and *TP53(−/−)* cells exposed to BaP and where results were confirmed by knocking down *TP53* in WT cells by siRNA (Hockley et al. 2008). Previous gene expression data suggested that off-target effects of the *TP53* knockout procedure by homologous recombination in HCT116 cells were minimal at the gene expression level (Hockley et al. 2008). Furthermore, the same phenomenon was observed in other cell lines (e.g. lung A549 cells) with stably knocked down p53 expression, although the difference in adduct levels in cells with no p53 expression was greater (Hockley et al. 2008). As this phenomenon was not observed after exposure to the PAH-diol-epoxides (compare Fig. 2), these results indicate that the levels of p53 expression impact on the metabolic activation of the parent PAH.

In the present study, we also showed that BaP metabolites measured in the culture media were higher with HCT116 *TP53(+/+)* cells than with *TP53(−/−)* cells (Table 1). The measured metabolites included BaP-tetrols, BaP-dihydrodiols and hydroxylated-BaP metabolites demonstrating that CYP-mediated metabolism was impaired in *TP53(−/−)* cells, which correlated with lower BaP-DNA adduct formation in these cells. Higher induction of *CYP1A1* expression (Fig. 3) and an increase in CYP1A1 protein levels (Fig. 1) in HCT116 cells with WT p53 explains the difference in BaP metabolism observed in *TP53(−/−)* cells. Furthermore, pre-treatment of HCT116 *TP53(+/+)* cells with nutlin-3a led to p53 induction and subsequent stimulation of CYP1A1 expression after co-exposure to BaP (Fig. 4a). Consequently, p53 induction by nutlin-3a enhanced CYP1A1-mediated BaP metabolism (Fig. 4b).

Many studies have demonstrated the induction of p53 in response to PAH exposure and this can be of significance for the molecular phenomenon (i.e. impact of *TP53* status on PAH-induced DNA adduct formation) described above. We and others previously identified p53 as an important component of PAH-induced transcriptional responses in human cell lines and in vivo (Hamouchene et al. 2011; Hockley et al. 2006, 2008; Labib et al. 2012; Malik et al. 2012, 2013). Thus, these gene–environment interactions need to be taken into account with regards to PAH metabolism. Furthermore, as many anti-cancer treatment regimens are composed of several drugs of which at least one is a p53 activating drug, our molecular observation may also be of clinical importance (Goldstein et al. 2013). Therefore, these potential drug-environmental interactions may also be carefully considered for human risk assessment.

A well-established molecular mechanism for the induction of CYP1A1 is via AHR with 2,3,7,8-tetrachlorodibenzo-p-dioxin (TCDD) being one of the best characterised ligands for this orphan nuclear receptor.(Wang et al. 2011) PAHs such as BaP can also bind to and activate AHR thereby enhancing their own metabolic activation (Hockley et al. 2007). However, a recent study has indicated that p53 can induce gene expression of certain *CYPs* by binding to p53REs in the promoter region of these *CYPs*. It was shown that p53 induces the activity of CYP3A4, one of the major drug-metabolising enzymes in the liver, via its binding to p53REs and the subsequent transcriptional enhancement of *CYP3A4* (Goldstein et al. 2013). In the present study, we propose a new mechanism by which CYP1A1 is induced by p53. Using ChIP analysis, we showed that p53 can bind to a p53RE in the regulatory region upstream of the *CYP1A1* gene in HCT116 *TP53(+/+)* cells after BaP exposure (Fig. 5). Consequently, these results indicate that BaP is able to induce CYP1A1 not only by binding as a ligand to AHR promoting *CYP1A1* transcriptional activation, but also by induction of p53 which can subsequently trigger *CYP1A1* transcription via its binding to p53REs.

Both complete and partial inactivation of tumour suppressors such as *TP53* can play a critical role in the pathogenesis of cancer (Berger et al. 2011). It has been shown that reductions in p53 dosage and function can impact on a cell’s ability to respond to DNA damage (Berger et al. 2011; Bunz et al. 1999). Deletions of the *TP53* locus are frequently found in human cancer, and loss of heterozygosity (LOH) has been shown to occur in a fraction of tumours harbouring a *TP53* mutation (Berger and Pandolfi 2011). *TP53* is a powerful example of how haploinsufficiency promotes tumourigenesis, whereby loss of one copy of *TP53* gives rise to a phenotype intermediate to that occurring after complete loss of the gene (Berger and Pandolfi 2011). As such, survival of *Trp53(*+*/−)* mice show an intermediate survival to that *Trp53(−/−)* and *Trp53(+/+)* mice, and tumours that develop in *Trp53(*+*/−)* mice do not always display loss of the remaining WT allele (Berger et al. 2011; Venkatachalam et al. 1998). Therefore, it was expected that HCT116 *TP53(*+*/−)* cells generate an intermediate response to those observed in *TP53(−/−)* and *TP53(+/+)* cells. Interestingly, HCT116 *TP53(*+*/−)* cells behaved more similar to *TP53(−/−)* cells than *TP53(+/+)* cells with regards to PAH-DNA adduct formation, while adduct formation induced by the corresponding PAH-diol-epoxides again did not differ (Fig. 2). This observation was in accordance with the fact that HCT116 *TP53(*+*/−)* and *TP53(−/−)* cells formed similar amounts of BaP metabolites (Table 1). Expression of CYP1A1, at both the gene and protein level (Figs. 1, 3), was also similar in both cell lines. These results suggest that in HCT116 *TP53(*+*/−)* cells, the remaining WT allele is not sufficient for normal cellular function. This possibility has been indicated in another study using these cells where loss of one WT allele resulted in a ~fourfold reduction in *TP53* mRNA and protein levels compared with *TP53(+/+)* cells before and after p53 stabilization resulting from UV radiation (Lynch and Milner 2006). One reason for this phenomenon could be that p53 function is dependent on its tetrameric structure and reduction in dosage of the WT protein by 50 % may result in disproportionate reduction in active tetramer concentrations (Santarosa and Ashworth 2004).

The importance of p53 is also highlighted by the fact that it is mutated in over 50 % of human cancers (Olivier et al. 2010). Mutation patterns and spectra in *TP53* have been linked to environmental exposures including PAHs (Arlt et al. 2007; Kucab et al. 2010). For example, lung tumours of tobacco smokers (but not of nonsmokers) contain a high percentage of G to T transversions in *TP53* at several hotspot locations (in particular, at codons 157, 158, 175, 245, 248 and 273) characteristic of PAHs present in tobacco smoke.(Denissenko et al. 1996) Of note, hotspot mutations found at codons 175, 248, 249 and 273 together account for over 25 % of all missense mutations identified in human cancers (Olivier et al. 2010). Mutant p53 expressed in preneoplastic and/or neoplastic cells can severely limit or abolish the capacity of p53 to regulate its target genes (Freed-Pastor and Prives 2012). One *TP53* hotspot mutation found in human tumours, R248 W, has been identified as a contact mutation that induces conformational changes and abolishes the tumour suppressive activity of p53 (Song et al. 2007). Both in HCT116 cells where one WT allele was replaced with mutant (R248 W) allele, and in cells where one WT allele was inactivated by homologous recombination and the other was replaced with a mutant (R248 W) allele, PAH metabolism and PAH-DNA adduct formation was altered relative to *TP53(+/+)* cells (Table 1; Figs. 1, 2, 3). The response in HCT116 cells with mutant p53 was similar to that in cells having a complete knockout of p53. However, it is possible that cells carrying a different *TP53* mutation may respond differently. A more comprehensive approach to studying the impact of distinct *TP53* mutations in cells with a closely matched genetic background for comparative functional analysis of p53 and carcinogen metabolism in the future has been published recently (Odell et al. 2013; Wei et al. 2011).

The responses seen after PAH exposure in HCT116 cells with altered p53 expression have been similar for different compounds in this study, but the response to other environmental carcinogens could be different. Another environmental carcinogen which has been tested in HCT116 *TP53(+/+)* and *TP53(−/−)* cells is aristolochic acid I (AAI), a herbal drug derived from the *Aristolochia* species which is a potent human carcinogen involved in the development of aristolochic acid nephropathy and linked to urothelial cancer (Gokmen et al. 2013; Schmeiser et al. 2014). The major activation pathway for AAI is nitroreduction, NQO1 being the most efficient activating enzyme, while CYP1A-mediated demethylation contributes to AAI detoxification (Stiborova et al. 2014). AAI-DNA adduct formation was significantly lower in *TP53(−/−)* relative to *TP53(+/+)* cells (Simoes et al. 2008). Interestingly, *CYP1A1* gene expression measured by qRT-PCR analysis was higher in *TP53(−/−)* compared with *TP53(+/+)* cells after AAI exposure (Simoes et al. 2008), suggesting that cellular *TP53* status may impact on CYP1A1-mediated AAI detoxification. However, the mechanism by which *TP53* status influences AAI metabolism and AAI-DNA adduct formation in human cells remains to be investigated further.

In contrast, DNA adduct formation by a nitroarene, 3-nitrobenzanthrone (3-NBA), was similar in HCT116 *TP53(+/+)* and *TP53(−/−)* cell lines (Hockley et al. 2008; Simoes et al. 2008). This observation indicates that the cellular impact of p53 on carcinogen metabolism depends on the agent studied and/or that only certain XMEs depend on p53 function. The most efficient enzyme to activate 3-NBA to DNA adducts is NQO1 (Arlt et al. 2005, 2006; Stiborova et al. 2010). Thus, NQO1 seems to be an enzyme not influenced by p53, which is in line with the fact that NQO1 protein expression was not altered in the HCT116 cell lines after BaP exposure (Fig. 2). Another recent study showed that induction of *CYP2E1* gene expression is under the transcriptional control of p53 (Leung et al. 2013). As CYP2E1 overexpression inhibited migration of highly invasive MDA-MB-231 breast cancer cells expressing mutant p53, the authors concluded that manipulation of CYP2E1 protein expression could be potentially exploited in breast cancer therapy (Leung et al. 2013). However, in the context of our investigations, this study indicates that CYP2E1-mediated carcinogen metabolism may be altered by p53 expression and seems to be a promising target for future investigations. One typical substrate of CYP2E1 is ethanol which is the main cause of various liver diseases (Beier and McClain 2010). Another recent study demonstrated that UDP-glucuronosyltransferase 2B7 (UGT2B7) expression is regulated via the p53 pathway (Hu et al. 2014). Epirubicin, an anticancer drug, stimulated *UGT2B7* promoter activity via p53RE in human hepatocarcinoma HepG2 cells and similarly nutlin-3a enhanced *UGT2B7* expression and recruited p53 protein to the *UGT2B7* p53RE in these cells (Hu et al. 2014). Collectively, these findings suggest a novel role for p53 in the regulation of both phase I and phase II XMEs.

In summary, we found that carcinogenic PAHs induce CYP1A1 expression in human cells via a p53-dependent mechanism. Thus, our identification of *CYP1A1* as novel p53 target gene provides new insights into the mechanism controlling *CYP1A1* expression. These results also provide new fundamental insights into the mechanism(s) of p53 in PAH-induced carcinogenesis. These results indicate that p53 may impact on the efficacy of chemotherapeutic drugs. However, they also indicate that gene–environment interactions need to be taken into account with regards to carcinogen metabolism. Future investigations will need to clarify which other XMEs depend on p53 function and which other environmental carcinogens depend on p53 for their metabolic activation.

## Electronic supplementary material

Below is the link to the electronic supplementary material.

Supplementary material 1 (DOC 281 kb)
