# Supplementary material for: Carcinogenic polycyclic aromatic hydrocarbons induce CYP1A1 in human cells via a p53-dependent mechanism
Source: Arch Toxicol. 2014 Nov 15;90(2):291–304. doi: 10.1007/s00204-014-1409-1 (PMC4748000; doi:10.1007/s00204-014-1409-1)
Supplement: Supplementary file 1 — Supplementary material 1 (DOC 281 kb) [file 204_2014_1409_MOESM1_ESM.doc]

**Supporting Figure 1**

Cytochrome P450-mediated metabolic activation and DNA adduct formation of (**A**) BaP, (**B**) DB[*a,h*]A and (**C**) DB[*a,l*]P. Scheme includes the chemical structures of the PAH test compounds and their corresponding diol-epoxides.

**Supporting Figure 2**

Effect of PAHs and their corresponding diol-epoxides on cell viability (% control) in isogenic HCT116 cells. Cells were exposed to (**A**) 2.5 μM BaP, (**B**) 0.5 μM BPDE, (**C**) 2.5 μM DB[*a,h*]A, (**D**) 0.5 μM DB[*a,h*]ADE, (**E**) 2.5 μM DB[*a,l*]P or (**F**) 0.0025 μM DB[*a,l*]PDE and harvested after the times indicated. Values are means ± SD of two separate incubations with two independent samples each (*n*=4). Statistical analysis was performed by one-way ANOVA followed by the Tukey post-hoc test (**p*<0.05, ***p*<0.01, different from HCT116 *TP53(+/+)* cells).

**Supporting Figure 3**

Effect of BaP on the cell cycle in HCT116 cells. Cell were exposed to 2.5 μM BaP and harvested after 24 (**A**) or 48 h (**B**). Values are means of two separate incubations with two samples each. Statistical analysis was performed by two-way ANOVA followed by the Tukey post-hoc test; **p*<0.05, different from BaP-treated HCT116 *TP53(+/+)* cells; *#p*<0.05, different from DMSO-treated HCT116 *TP53(+/+)* cells).

**Supporting Figure 4**

BaP metabolism catalysed by cytochrome P450 (CYP) 1A1. Scheme shows the chemical structures of BaP metabolites analysed by HPLC analysis (see Table 1).

**Supporting Figure 5**

Repair capacity of extracts from HCT116 *TP53(+/+)*,*TP53(+/-)*, *TP53(-/-)*, *TP53(R248W/+)* and *TP53(R248W/-)* cells. Values are means ± SD of three separate experiments. Statistical analysis was performed by one-way ANOVA followed by the Tukey post-hoc test; no significant differences were observed.
